# LGR5 promotes cancer stem cell traits and chemoresistance in cervical cancer

**DOI:** 10.1038/cddis.2017.393

**Published:** 2017-09-07

**Authors:** Hao-Zhe Cao, Xiao-Fang Liu, Wen-Ting Yang, Qing Chen, Peng-Sheng Zheng

**Affiliations:** 1 Department of Reproductive Medicine, the First Affiliated Hospital, Xi’an Jiaotong University Medical School, Xi’an 710061, China; 2 Department of Pharmacology, Shannxi Provincial Tumor Hospital, Xi’an 710061, China; 3 Department of Obstetrics and Gynecology, the Second Affiliated Hospital, Xi’an Jiaotong University Medical School, Xi’an 710061, China; 4 Division of Cancer Stem Cell Research, Key Laboratory of Environment and Genes Related to Diseases, Ministry of Education, Xi’an Jiaotong University Medical School, Xi’an 710061, China

## Abstract

Cancer stem cells (CSCs), also known as tumor-initiating cells, contribute to tumorigenesis, resistance to chemoradiotherapy and recurrence in human cancers, suggesting targeting CSCs may represent a potential therapeutic strategy. Leucine-rich repeat-containing G-protein-coupled receptor 5 (LGR5) has recently been found to be a bona fide marker of colorectal CSCs. Our previous study showed that LGR5 functions as a tumor promoter in cervical cancer by activating the Wnt/*β*-catenin pathway. However, very little is known about the function or contribution of LGR5 to cervical CSCs. Here, we have modulated the expression of LGR5 using an overexpression vector or short hairpin RNA in cervical cancer cell lines. We demonstrated that elevated LGR5 expression in cervical cancer cells increased tumorsphere-forming efficiency; conferred chemoresistance to cisplatin treatment; augmented cell migration, invasion and clonogenicity; and elevated the levels of stem cell-related transcription factors *in vitro*. Furthermore, modulated LGR5^+^ cells, unlike LGR5^−^ cells, were highly tumorigenic *in vivo*. In addition, the modulated LGR5^+^ cells could give rise to both LGR5^+^ and LGR5^−^ cells *in vitro* and *in vivo*, thereby establishing a cellular hierarchy. Finally, we found that the increased tumorsphere-forming efficiency induced by LGR5 could be regulated through the inhibition or activation of the Wnt/*β*-catenin pathway in cervical cancer cells. Taken together, these results indicate that LGR5 has a vital oncogenic role by promoting cervical CSC traits and may represent a potential clinical target.

Cervical cancer is the third most common type of malignant tumor and the fourth leading cause of cancer death among women worldwide.^1, 2^ Cervical cancer development begins with the infection of the cervical epithelium by high-risk human papillomaviruses.^3^ Although cervical cancer can be detected in its early stages by HPV testing and Papanicolaou smear screening and successfully eradicated through surgery, curative treatments do not yet exist for advanced, recurrent or metastatic cervical cancer.^4^ Previous studies have suggested that only a rare subpopulation of tumor cells called cancer stem cells (CSCs) can regenerate the tumor and may be involved in therapy resistance, tumor relapse and metastasis.^5^ Therefore, more effective tumor therapies require a better understanding of the characteristics of this subset of cancer cells and the factors, both extrinsic and intrinsic, which contribute to their existence or stemness. Our previous studies have demonstrated that high aldehyde dehydrogenase activity may be used to identify CSCs in human cervical cancer.^6^ Recently, a number of studies have found that several stem cell-related genes are closely associated with tumorigenesis, and it has been demonstrated that SOX2,^7^ NANOG,^8^ KLF4,^9^ OCT4^10^ and LGR5^11^ have critical roles in cervical carcinogenesis.

The leucine-rich repeat-containing G-protein-coupled receptor 5 (LGR5), also known as GPR49, belongs to the G-protein-coupled receptor family of proteins and is a target gene of Wnt/*β*-catenin signaling. LGR5 has been identified as a novel marker of adult stem cells in the small intestine and hair follicles.^12, 13, 14^ LGR5 also has an important role during embryogenesis. In recent years, many studies have revealed that LGR5 is overexpressed in various types of tumors, including colorectal cancer,^15^ ovarian tumors,^16^ hepatocellular carcinoma,^17^ basal cell carcinoma^18^ and esophageal adenocarcinoma.^19^ In addition, LGR5 has been recognized as a CSC marker for colorectal cancers.^20^ Our previous study showed that LGR5 was progressively expressed in cervical carcinogenesis and promoted the proliferation of cervical cancer cells as well as tumor formation by potentiating the Wnt/*β*-catenin pathway.^11^ Thus, we hypothesized that LGR5 might contribute to cervical carcinogenesis, recurrence and metastasis by maintaining the stemness of cervical CSCs.

In this study, we used standard functional assays and fluorescence activated cell sorting (FACS) to analyze the properties of cervical CSCs with different levels of LGR5 expression. The results indicated that a subpopulation of human cervical cancer cells with elevated LGR5 expression possesses enhanced self-renewal capacity, differentiation potential and tumorigenicity. Furthermore, we found that the increased tumorsphere-forming efficiency induced by LGR5 could be regulated through the inhibition or activation of the Wnt/*β*-catenin pathway in cervical cancer cells. Our data indicated that LGR5 has a vital oncogenic role through promoting CSC traits in cervical cancer.

## Results

### Elevated LGR5 enhances the self-renewal capacity of cervical cancer cells

**Figure 1:**
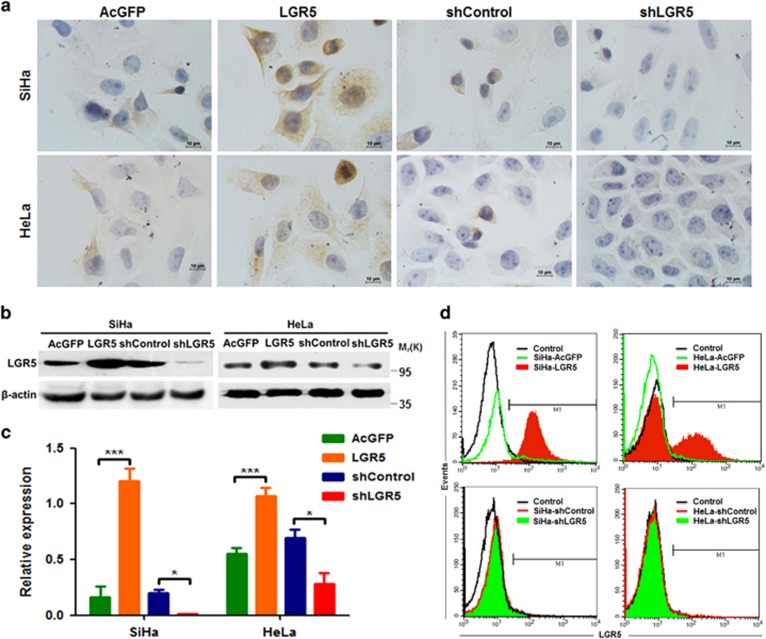
Overexpression and knockdown of LGR5 in human cervical cancer cell lines. (**a**) Immunohistochemical staining showing LGR5 expression in LGR5-overexpressing and LGR5-knockdown SiHa and HeLa cells, scale bar, 10 *μ*m. (**b**) A western blot assay was used to characterize the expression of LGR5 in LGR5-overexpressing and LGR5-knockdown SiHa and HeLa cells. (**c**) The expression of LGR5 in HeLa, SiHa cells was measured by western blot. (**d**) LGR5 expresssion was analyzed by flow cytometry. AcGFP: green fluorescent protein for control; LGR5: overexpression for LGR5; shLGR5: shRNA for LGR5; shControl: shRNA for control. Values are shown as the mean±S.D. *P<0.05; ***P<0.001

As a CSC marker, LGR5 has been shown to be progressively expressed in cervical carcinogenesis and to promote cancer cell proliferation and tumor formation. To further investigate the mechanism involved in tumor promotion by LGR5 in cervical cancer, stable LGR5-overexpressing cells (SiHa-LGR5 and HeLa-LGR5) and stable LGR5-knockdown cells (SiHa-shLGR5 and HeLa-shLGR5) were established in cervical cancer cell lines. The expression of LGR5 in these cell lines was examined by immunohistochemistry (IHC), western blot and flow cytometry. As shown in Figure 1a, high expression of LGR5 was detected in SiHa-LGR5 and HeLa-LGR5 cells within the cell cytoplasm and membrane; LGR5 was almost undetectable in LGR5-silenced cells. Western blot analysis showed the semi-quantitative expression of LGR5 relative to *β*-actin was significantly higher in LGR5-overexpressed cells and lower in LGR5-silenced cells compared with control cells (*P*<0.05, Figures 1b and c). To confirm this, flow cytometry was used to assess the expression of LGR5. The LGR5-positive cell population was detected in 1.7% of the SiHa-AcGFP cells, 80.1% of the SiHa-LGR5 cells, 1.7% of the SiHa-shControl cells and 0.8% of the SiHa-shLGR5 cells. Similarly, the LGR5-positive population was detected in 1.3% of the HeLa-AcGFP cells, 35.6% of the HeLa-LGR5 cells, 1.3% of the HeLa-shControl cells and 0.5% of the HeLa-shLGR5 cells (Figure 1d).

**Figure 2:**
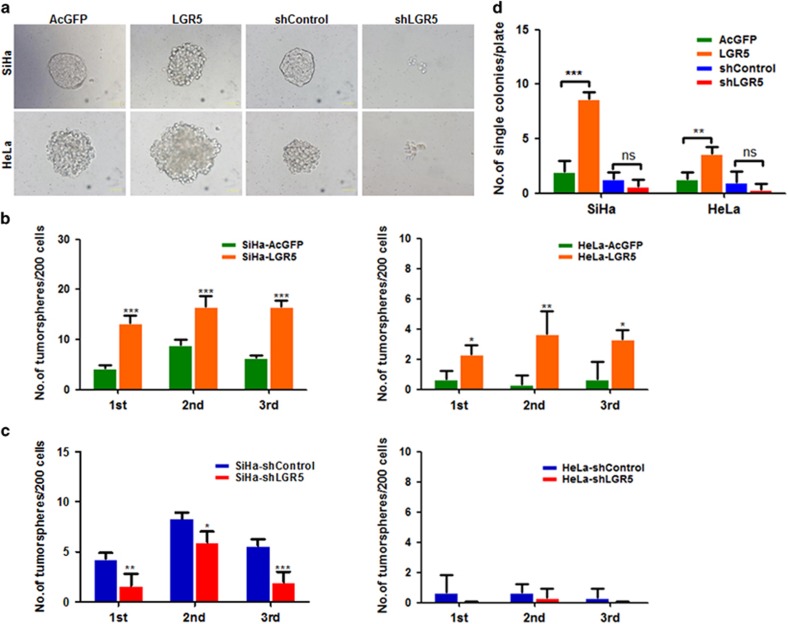
LGR5-overexpressing cervical cancer cells exhibit enhanced self-renewal capacity. (**a**) Representative photos of tumorspheres formed by LGR5-overexpressing and LGR5-knockdown SiHa and HeLa cells. Bar, 50 *μ*m. (**b**, **c**) The number of tumorspheres/200 cells was counted from three consecutive passages. (**d**) The number of wells containing tumorspheres was counted. *P<0.05; **P<0.01; ***P<0.001. Data represent mean±S.D. of triplicate experiments

To investigate the role of LGR5 on the self-renewal capacity of cervical cancer cells, which is one of the most important characteristics of CSCs, LGR5-overexpressing cells (SiHa-LGR5 and HeLa-LGR5) and LGR5-knockdown cells (SiHa-shLGR5 and HeLa-shLGR5), as well as control cells, were cultured in serum-free medium under conditions that are optimal for growing tumorspheres. As shown in Figure 2a, LGR5-overexpressing cells and control cells generated typical tumorspheres, whereas LGR5-knockdown cells did not form any tumorspheres but formed only a few cell aggregates. To compare the tumorsphere-formation capacity, 200 cells/well were plated onto 24-well plates and then cultured for three passages in conditioned medium. We found that LGR5-overexpressing cells formed 2.0~4.5-fold more tumorspheres than control cells in both the SiHa and HeLa cell lines (*P*<0.05, Figure 2b). Depletion of LGR5 reduced the spheroid formation efficiency by 25–60% in SiHa cells, whereas the HeLa-shLGR5 cells rarely formed tumorspheres or did not form any tumorspheres (Figure 2c). To exclude the effects of cell aggregation, which can occur in low-density cultures, one cell was cultured in each well by limited dilution. The SiHa-LGR5 and HeLa-LGR5 cells generated tumorspheres with an efficiency of 8.7% and 3.6%, respectively, whereas the SiHa-AcGFP and HeLa-AcGFP cells generated tumorspheres with an efficiency of 2.0% and 1.3%, respectively. Furthermore, the SiHa-shLGR5 and HeLa-shLGR5 cells generated tumorspheres with an efficiency of 0.7% and 0.3%, respectively, whereas the SiHa-shControl and HeLa-shControl cells generated tumorspheres with an efficiency of 1.3% and 1.0%, respectively (Figure 2d). These data indicate that elevated LGR5 expression enhances the self-renewal capacity of cervical cancer cells.

### Elevated LGR5 expression enhances the tumorigenicity of cervical cancer cells *in vivo*

**Figure 3:**
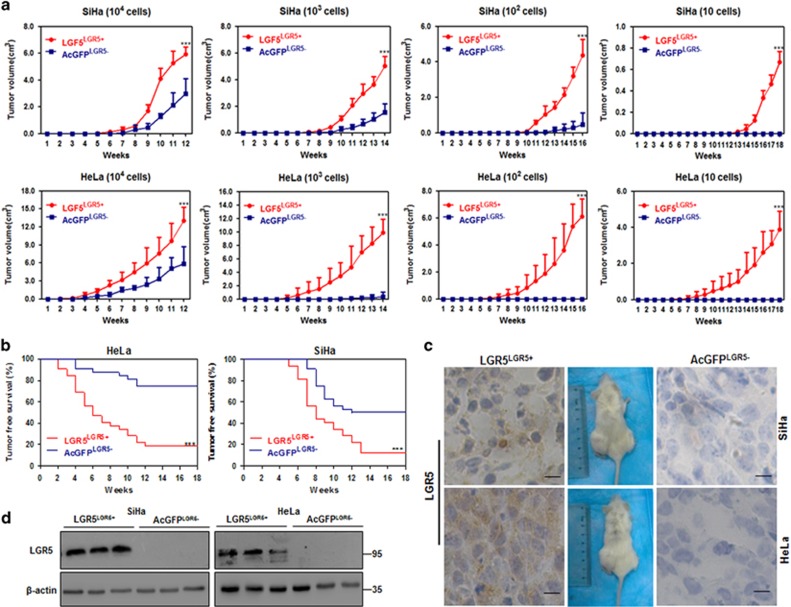
Tumorigencity of LGR5^LGR5+^ and AcGFP^LGR5^^–^ cells from two cervical cancer cell lines in NOD/SCID mice. (**a**) The volume of xenograft tumors formed by different numbers of LGR5^LGR5+^ and AcGFP^LGR5−^ cervical cancer cells was monitored over time. (**b**) Kaplan–Meier plots showing the tumor-free survival after injection. (**c**, **d**) Immunohistochemical staining and western blot for LGR5 in tumor tissues, scale bar, 10 *μ*m. *P<0.05; **P<0.01; ***P<0.001. Data represent mean±S.D. of tumor volumes at different time points of eight mice in each group

One of the most important characteristics of CSCs is their powerful ability to form tumors in xenografts. To determine whether upregulated LGR5 could greatly enhance the tumorigenic capacity of cervical cancer cells, the LGR5^+^ and LGR5^–^ cell populations were sorted and purified, respectively, from LGR5-overexpressing cells and LGR5-AcGFP cells, and then, different doses of cells were subcutaneously injected into the flanks of NOD/SCID mice by limiting dilutions. First, the tumor volume was monitored twice a week, and the results are shown in Figure 3a. Both LGR5^+^ and LGR5^–^ SiHa cells administered at the dose of 10^4^, 10^3^ or 10^2^ cells led to tumor formation. However, the tumors formed by the LGR5^+^ SiHa-LGR5 cells were larger and grew faster than those formed by the LGR5^–^ SiHa-AcGFP cells (*P*<0.05). Furthermore, the LGR5^+^ SiHa-LGR5 cells were capable of tumor formation at a dose of 10 cells, but the LGR5^–^ SiHa-AcGFP cells could not (Figure 3a, panel 1). Upon inoculation with 10^4^ or 10^3^ LGR5^+^ and LGR5^–^ modified HeLa cells, the LGR5^+^ HeLa-LGR5 population more rapidly formed larger palpable tumors than the LGR5^–^ HeLa-AcGFP population. However, when inoculated with 10^2^ or 10 cells, the LGR5^+^ HeLa-LGR5 cells, but not the LGR5^–^ HeLa-AcGFP cells, were capable of forming palpable tumors (Figure 3a, panel 2).

Tumor latency was monitored after the injection of sorted cells into NOD/SCID mice and was defined the tumor-free duration in the mice (Figure 3b). Inoculation with LGR5^+^ SiHa-LGR5 cells led a significantly shorter tumor-free period; for instance, the shortest tumor-free period following LGR5^+^ SiHa-LGR5 cell implantation was 5 weeks, compared with the 7-week tumor-free period exhibited by mice inoculated with LGR5^–^ SiHa-AcGFP cells. During 18 weeks, LGR5^+^ SiHa-LGR5 cells also caused a lower tumor-free rate (12.5% for LGR5^+^ SiHa-LGR5 cells versus 50% for LGR5^–^ SiHa-AcGFP cells) than LGR5^–^ SiHa-AcGFP cells (*P*<0.01). Similarly, the LGR5^+^ HeLa-LGR5 cells were associated with a significantly shorter tumor-free period (2 weeks for LGR5^+^ HeLa-LGR5 cells versus 4 weeks for LGR5^–^ HeLa-AcGFP cells) and a lower tumor-free rate (18.75% for LGR5^+^ HeLa-LGR5 cells versus 75% for LGR5^–^ HeLa-AcGFP cells) compared with the LGR5^–^ HeLa-AcGFP cells (*P*<0.001). The cervical CSC frequency in the LGR5^+^ and LGR5^–^ modified cervical cancer cell populations is summarized in Table 1. The frequency of tumor-initiating LGR5^+^ SiHa-LGR5 cells was 1/36, which was 17.4-fold higher than that of the LGR5^–^ SiHa-AcGFP cells (1/627; *P*<0.001). The frequency of tumor-initiating LGR5^+^ HeLa-LGR5 cells (1/46) was 136.4-fold higher than that of the LGR5^–^ HeLa-AcGFP cells (1/6,276; *P*<0.001). In addition, LGR5 protein expression remained high in LGR5^+^ cells and was not detectable in LGR5^–^ cells within the tumor xenografts tissues as determined by immunohistochemical staining and western blot (Figures 3c and d).

Together, these results from the tumor formation assays in NOD/SCID mice suggest that cervical cancer cells with elevated LGR5 expression have a more rapid tumor growth rate, shorter tumor latency, lower tumor-free rate and higher frequency of tumor-initiating cells than LGR5-negative cells. Therefore, elevated LGR5 expression could enhance the tumorigenic capacity of cervical cancer cells *in vivo.*

### LGR5-positive cells have the ability to differentiate *in vitro* and *in vivo*

**Figure 4:**
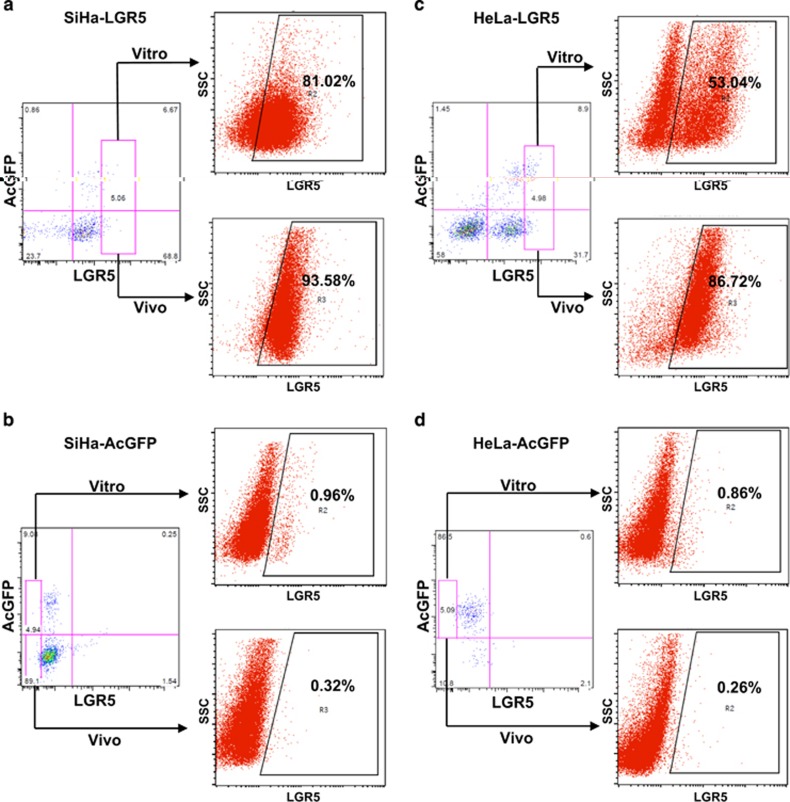
LGR5^+^ cervical cancer cells are capable of differentiating *in vitro* and *in vivo*. (**a**–**d**) LGR5^+^ and LGR5^−^ cells were isolated from LGR5-overexpressing SiHa and HeLa or control cells and cultured in DMEM medium supplemented with 10% FBS and 1000 *μ*g/ml G418 for 2 weeks *in vitro*. The xenograft tumor cells from LGR5^+^ and LGR5^−^ cells *in vivo* were digested by collagenase IV overnight before detection. The expression of LGR5 was analyzed by flow cytometry

One characteristic of CSCs is the capacity to differentiate into non-CSCs and give rise to heterogeneous tumor cell populations. To determine whether the modulated LGR5-positive cells were capable of differentiation *in vitro*, LGR5^+^ and LGR5^–^ cell populations were cultured separately in DMEM supplemented with 10% fetal bovine serum (FBS) and 1000 *μ*g/ml G418 for 2 weeks. After incubation, the cultured populations were analyzed by flow cytometry. Approximately 19.0% of the modulated LGR5^+^ SiHa-LGR5 cells differentiated into LGR5^–^ SiHa-LGR5 cells, and 81.0% of these cells remained LGR5-positive (Figure 4a, upper panel). However, >99.0% of the LGR5^–^ SiHa-AcGFP cells retained the phenotype (Figure 4b, upper panel). Similarly, 47.0% of the modulated LGR5^+^ HeLa-LGR5 cells generated LGR5^–^ HeLa-LGR5 cells (Figure 4c, upper panel). However, 99.1% of the LGR5^–^ HeLa-AcGFP cells maintained the LGR5-negative phenotype (Figure 4d, upper panel).

The differentiation capacity of the modulated LGR5-positive cells and LGR5-negative cells was also assessed *in vivo*. In the tumors formed by LGR5^+^ SiHa-LGR5 cells, a few LGR5^–^ SiHa-LGR5 cells and many LGR5^+^ SiHa-LGR5 cells were found, indicating that LGR5^+^ SiHa-LGR5 cells were able to generate LGR5^+^ SiHa-LGR5 cells through self-renewal and to generate LGR5^–^ SiHa-LGR5 through differentiation (Figure 4a, lower panel). However, in the tumors formed by LGR5^–^ SiHa-AcGFP cells, no LGR5^+^ SiHa-AcGFP cells were found, indicating that LGR5^–^ SiHa-AcGFP cell did not have the ability to differentiate (Figure 4b, lower panel). Similarly, 13.3% of the LGR5^+^ HeLa-LGR5 cells generated LGR5^–^ HeLa-LGR5 cells, and 86.7% of the cells remained LGR5-positive (Figure 4c, lower panel). However, 99.7% of the LGR5^–^ HeLa-AcGFP cells maintained the LGR5-negative phenotype (Figure 4d, lower panel). Taken together, these data demonstrate that the LGR5-positive cells from LGR5-overexpressing cervical cancer cells have the ability to differentiate both *in vitro* and *in vivo*.

### Elevated LGR5 expression protects cervical cancer cells from cisplatin-induced cell death

**Figure 5:**
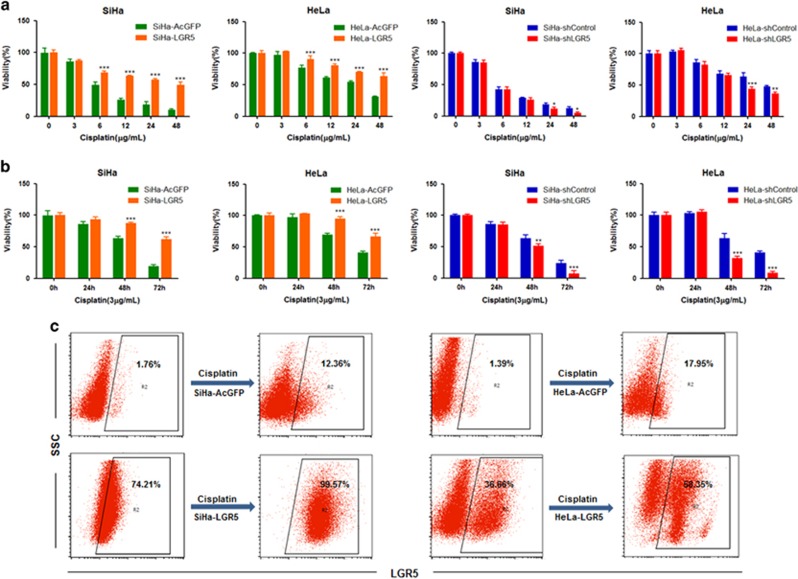
Elevated LGR5 expression protects cervical cancer cells to resist cisplatin. (**a**) Cell viability of the LGR5-overexpressing and LGR5-knockdown cervical cancer cells was measured using an MTT assay after treatment with different concentrations of cisplatin for 24 h. (**b**) Cell viability of the LGR5-overexpressing and LGR5-knockdown cervical cancer cells was measured using an MTT assay after treatment with a constant dose of cisplatin for 0, 24, 48 or 72 h. Data represent mean±S.D. of triplicate experiments. (**c**) The percentage of LGR5^+^ cells in the cervical cancer cell lines was analyzed by flow cytometry after exposure to 3 *μ*g/ml cisplatin for 2 days and following culture in ordinary culture medium for 2 weeks. *P<0.05; **P<0.01; ***P<0.001

The chemotherapeutic resistance of CSCs is thought to be responsible for cancer recurrence and metastasis.^21^ Because cisplatin is one of the most commonly used chemotherapeutic drugs in the treatment of cervical cancer, we tested the effects of cisplatin on cervical cancer cells with different expression levels of LGR5. These cells were exposed to different concentrations of cisplatin for 24 h, and cell viability was determined using an MTT assay. The viability of both LGR5-overexpressing and LGR5-knockdown cells from the SiHa and HeLa cells showed dose-dependency when cells treated with cisplatin (Figure 5a). Both SiHa-LGR5 and HeLa-LGR5 cells were significantly more resistant to cisplatin at concentrations ⩾6 *μ*g/ml than the control cells (SiHa-AcGFP and HeLa-AcGFP). In contrast, the viability of the SiHa-shLGR5 and HeLa-shLGR5 cells was significantly decreased compared with the control cells (SiHa-shControl and HeLa-shControl) when exposed to ⩾24 *μ*g/ml cisplatin. These results indicated that elevated LGR5 expression could enhance the resistance of cervical cancer cells to the proper concentration of cisplatin for a limited period of time.

Cell viability was also determined by the MTT assay after exposure to a constant concentration of cisplatin for 24, 48 or 72 h (Figure 5b). Both SiHa-LGR5 and HeLa-LGR5 cells were significantly more resistant to ⩾48 h of treatment with 3 *μ*g/ml cisplatin than the control cells. In contrast, the viability of the SiHa-shLGR5 and HeLa-shLGR5 cells was significantly decreased compared with the control cells when exposed to cisplatin treatment for ⩾48 h. The results indicate that elevated LGR5 expression could enhance the resistance of cervical cancer cells to a constant concentration of cisplatin for a certain period of time.

To further confirm whether the LGR5-positive cells were more resistant to cisplatin than LGR5-negative cells, we treated SiHa-AcGFP and HeLa-LGR5 cells with 3 *μ*g/ml cisplatin for 2 days and then cultured the cells in regular culture medium for 2 weeks. The percentage of LGR5^+^ cells expanded from 1.76% to 12.36% in the SiHa-AcGFP cell population, 74.21% to 99.57% in the SiHa-LGR5 cell population, 1.39% to 17.95% in the HeLa-AcGFP cell population and 36.66% to 58.35% in the HeLa-LGR5 cell population (Figure 5c). These data suggest that LGR5 may confer a survival advantage to cultured cervical cancer cells and enhance their resistance to cisplatin treatment.

### Elevated LGR5 expression enhances the migration, invasion and colony formation ability of cervical cancer cells *in vitro*

**Figure 6:**
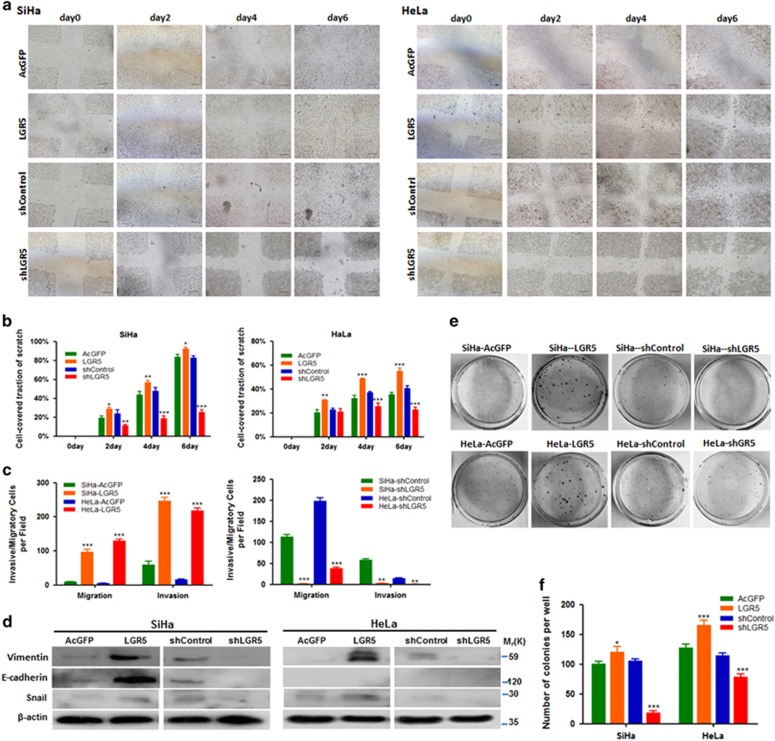
Elevated LGR5 enhances migration, invasion and colony formation ability of cervical cancer cells *in vitro.* (**a**, **b**) The overexpression of LGR5 enhances migration in wound healing assays. Columns and error bars represent means±S.D. from one experiment of three independent experiments (*n*=6 scratches per cell type and time point in each experiment). Scale bars, 800 *μ*m. (**c**) Cell migration and invasion was measured in Transwell chambers. Cells were counted with a microscope in nine random high-power fields. Migration and invasion ability was significantly increased in LGR5-overexpressing SiHa and HeLa cells. (**d**) Expression levels of EMT-related proteins in the LGR5-overexpressing and LGR5-knockdown HeLa and SiHa are demonstrated by western blotting. (**e**, **f**) Each 1000 cells were cultured in soft agar plates for 2 weeks. The number of colonies was assessed using crystal violet staining. Columns and error bars represent means±S.D. of three independent experiments using triplicate measurements in each experiment. *P<0.05; **P<0.01; ***P<0.001

To evaluate the influence of LGR5 on cell migration and invasion, we performed wound healing and Transwell assays. In the wound healing assay, the LGR5-overexpressing SiHa and HeLa cells tended to cover a larger area of the initial scratch than their respective control cells at all time points, with statistically significant differences observed after 2 days. The SiHa and HeLa-shLGR5 cells tended to cover a smaller area of the initial scratch than their respective control cells at all time points, with statistically significant differences observed after 2 days and 4 days, respectively (Figures 6a and b). To reduce the impact of proliferation as a confounding variable, we additionally monitored the migratory and invasive ability through transwell membranes in each group and observed a significantly larger number of migratory and invasive LGR5-overexpressing cells compared with control cells after incubating for 24 h. Consistently, a significantly smaller number of migratory and invasive shLGR5 cells was detected compared with the control cells after incubating for 48 h (Figure 6c). Furthermore, the EMT-related factors E-Cadherin, Vimentin and Snail were highly expressed in LGR5-overexpressing SiHa cells as determined by western blot. Inhibition of LGR5 in SiHa cells led to the down-regulation of E-Cadherin, Vimentin and Snail. In addition, LGR5 overexpression in HeLa cells obviously increased the expression of Vimentin and Snail, whereas inhibition of LGR5 in HeLa cells led to the down-regulation of Vimentin and Snail (Figure 6d).

In the colony formation assay, both LGR5-overexpressing SiHa and HeLa cells formed significantly more and larger colonies compared with control cells, whereas both shLGR5 SiHa and HeLa cells formed significantly fewer and smaller colonies compared with control cells (Figures 6e and f). These data suggest that elevated LGR5 expression enhances migration, invasion and colony formation abilities and CSC-like characteristics.

### Elevated LGR5 expression promoted stemness through stem cell-related transcription factors and the Wnt/*β*-catenin pathway in cervical cancer cells

**Figure 7:**
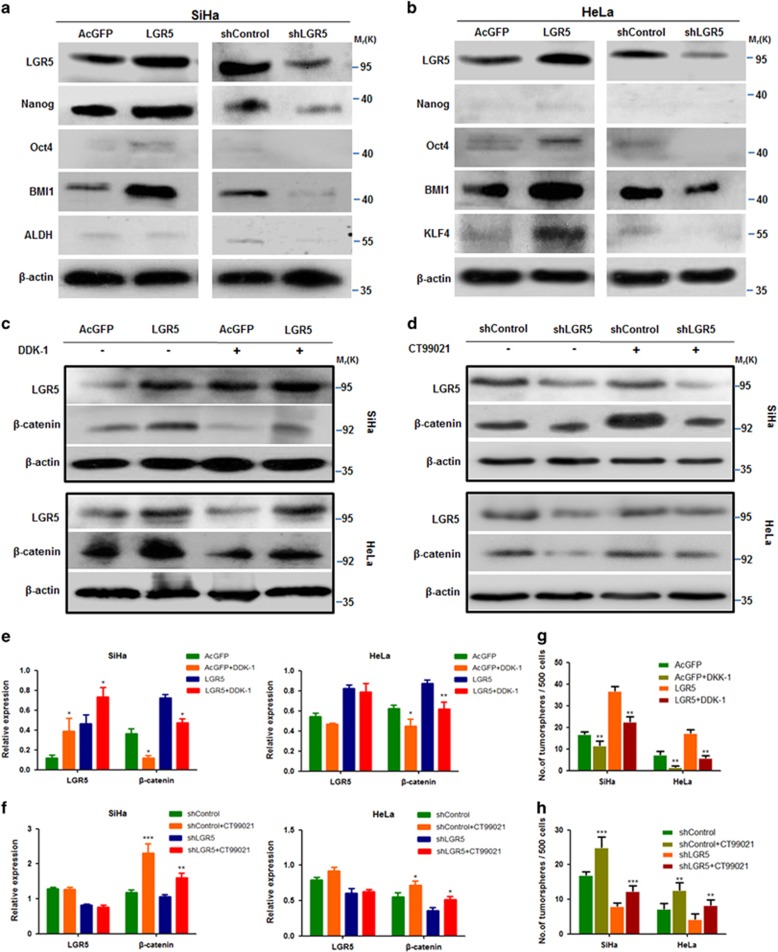
Elevated LGR5 promoting cervical cancer cell stemness associated with the Wnt/*β*-catenin pathway. (**a**, **b**) Western blot analysis of the protein levels of the stem cell-associated transcription factors OCT4, NANOG, KLF4, ALDH and BMI1 in the LGR5-overexpressing and LGR5-knockdown cervical cancer cells. *β*-actin was used as a loading control. (**c**–**f**) The expression of *β*-catenin in LGR5-overexpressing cervical cancer cell lines and control cells cultured with or without DDK-1 or CHIR-99021. (**g**, **h**) The tumorsphere-forming efficiency in modified cervical cancer cells was evaluated after change of Wnt/*β*-catenin pathway. Values are shown as the mean±S.D. of three experiments in duplicate. *P<0.05, **P<0.01; ***P<0.001 *versus* control using One-Way ANOVA

Stem cell-related transcription factors are important for maintaining the self-renewal capacity of embryonic stem cells. To clarify whether elevated LGR5 expression could promote the expression of stem cell-related transcription factors in cervical cancer cells, western blot analysis was performed to assess the expression of OCT4, NANOG, KLF4, ALDH and BMI1 in these cells. LGR5-overexpressing SiHa cells were found to express higher levels of BMI1, and have slight increase in NANOG and OCT4 expression than control cells (Figure 7a). LGR5-overexpressing HeLa cells were found to express higher levels of BMI1 and KLF4 than control cells (Figure 7b). Consistently, the expression levels of stem cell-associated transcription factors were reduced in the LGR5-knockdown cells. These data indicate that elevated LGR5 expression promotes a nuclear stemness signature in cervical cancer cells.

We have previously reported that LGR5 enhanced the proliferation and tumor formation abilities of cervical cancer cells by activating the Wnt/*β*-catenin signaling pathway.^11^ To understand whether elevated LGR5 expression is able to enhance cervical cancer cell stemness through Wnt/*β*-catenin signaling, DKK-1, an inhibitor of the Wnt/*β*-catenin pathway by binding to LRP6,^22^ was used to block the Wnt/*β*-catenin pathway in LGR5-modulated SiHa and HeLa cells. The expression of *β*-catenin was detected by western blot (Figures 7c and e). In the sphere formation assay, the tumorsphere-forming efficiency of the LGR5-overexpressing SiHa and HeLa cells with DKK-1 treatment was significantly decreased compared with those without DKK-1 treatment (Figure 7g). To further confirm that the Wnt/*β*-catenin pathway may be associated with the role of LGR5 in cancer stemness, CHIR-99021 (CT99021), an inhibitor of GSK-3*β* that suppresses *β*-catenin degradation, was used to activate the Wnt/*β*-catenin pathway in LGR5-knockdown SiHa and HeLa cells. The expression of *β*-catenin was also detected by western blot (Figures 7d and f). The tumorsphere-forming efficiency of the LGR5-knockdown SiHa and HeLa cells with CHIR-99021 treatment significantly increased compared with those without CHIR-99021 treatment (Figure 7h).

Taken together, these results suggested that the Wnt/*β*-catenin pathway is involved in the promotion of cervical cancer cell stemness by LGR5.

## Discussion

Despite being monoclonal in origin, most tumors appear to contain a heterogeneous population of cancer cells. According to the CSC hypothesis, among these heterogeneous cell populations, only a very small subpopulation of cancer cells, CSCs, possess enhanced self-renewal capacity, differentiation potential, tumorigenicity and chemoresistance. Previous studies have documented that purified LGR5^+^ cells exhibit stem cell properties, and LGR5 has been widely accepted as a marker for tumor-initiating cells in colorectal cancer and epithelial ovarian cancer.^23, 24^ In the present study, first, we found that forced expression of LGR5 remarkably increased spheroid formation capacity in SiHa and HeLa cells. The difference in the tumorspheroid formation rate of modified HeLa cells with depleted endogenous LGR5 was not statistically significant compared with that in the control groups (*P*>0.05), which might be because of the low spheroid formation capacity and low LGR5 expression level in HeLa cells. Second, the elevated expression of LGR5 was associated with an increase in stem cell marker expression while depletion of LGR5 reduced the expression of stem cell markers, such as OCT4, Nanog, Bmi-1 and KLF4 (Figure 7a), all of which have been associated with cancer stemness or been used to isolate CSC subpopulations in *vitro* and *in vivo*.^25, 26, 27, 28^ These results indicated that LGR5 positively modulates the expression of stem cell markers and stem-like properties in cervical cancer.

To understand the role of LGR5 in cervical tumorigenesis, which is a functional criterion for CSCs, LGR5^+^ cells were isolated from two modified cervical cancer cell lines. LGR5^+^ cervical cancer cells exhibited highly tumorigenic capacity *in vivo*. In addition, we found that modulated LGR5-positive cells could differentiate and re-establish the cellular hierarchy *in vitro* and *in vivo* (Figure 4). The modulated LGR5^+^ cells differentiated more slowly *in vivo* than that *in vitro*. This may be because that LGR5^+^ cells are more tumorigenic and easy to survive *in vivo*. These data indicated that alterations in the expression of specific cellular genes may alter the cell function and lineage status. In the other word, modified LGR5^+^ cells are indeed CSCs in cervical cancer.

Our results showed that forced expression of LGR5 was associated with increased cell migration, cell invasion and colony formation as well as enhanced chemoresistance *in vitro*. Moreover, depleting LGR5 decreased cell migration, cell invasion, colony formation and chemoresistance. This is consistent with previous studies of LGR5 in various cancers including basal cell carcinoma, gastric cancer, glioblastoma and colorectal carcinomas.^16, 20, 29, 30^ Sun *et al.*^31^ reported that LGR5 expression was associated with poor clinical survival of patients with cervical cancer, especially Stage II patients, indicating that high LGR5-expressing cells might be more aggressive and progress more quickly. This might be explained by our study, which showed that LGR5 promotes tumor progression by increasing the number of CSCs in the cervical cancer cell population that are associated with increased cell migration, cell invasion and chemoresistance capability. Also, this prompts us that LGR5 may be used as a potential therapeutic target for the treatment of cervical carcinoma with high expressing of LGR5.

In addition, we found that Wnt signaling was involved in the LGR5-associated cancer stemness in cervical cancer cells. The Wnt/*β*-catenin pathway is an ancient and highly conserved system that plays critical roles in the regulation of stem and CSCs.^32, 33^ In this study, elevated LGR5 expression promoted the expression of *β*-catenin in LGR5-modulated SiHa and HeLa cells. This is consistent with previous studies that LGR5 was found to potentiate Wnt/*β*-catenin signaling in HEK293T cells and in Ewing sarcoma.^34, 35^ Furthermore, the self-renewal capacity of cervical cancer cells was decreased by DKK-1 or increased by CHIR-99021 (Figure 7). These results suggest that the Wnt/*β*-catenin pathway is involved in the process by which LGR5 promotes cervical cancer cell stemness.

In summary, our comprehensive functional analysis of LGR5 in cervical cancer cell lines conclusively links LGR5 expression to cervical CSCs. LGR5 protein levels were found to be positively correlated with enhanced self-renewal capacities, differentiation potential and tumorigenicity; conferring chemoresistance; augmented cell migration, invasion and clonogenicity; and high levels of stem cell-related transcription factors. Wnt/*β*-catenin pathway may be involved in the process by which LGR5 promotes cervical CSC traits. Based on this study, LGR5 may be used as a potential therapeutic target for the treatment of cervical carcinoma.

## Material and methods

### Cell lines and culture conditions

Human cervical carcinoma cell lines HeLa and SiHa were purchased from the American Type Culture Collection (Manassas, VA, USA) and cultured in Dulbecco Modified Eagle Medium (Sigma-Aldrich, St Louis, MO, USA), supplemented with 10% FBS (Invitrogen, Carlsbad, CA, USA), penicillin and streptomycin.

### Vector construction and transfection

Human full-length LGR5 cDNA was amplified by reverse transcription polymerase chain reaction using mRNA extracted from SW620 cells. The primer sequences were designed as follows:

F5′-CTTCTCGAGCTACTTCGGGCACCATGG AC-3′ and

R5′-GCGGGTACCTTAGAGACATGGGACAAA TG-3′.

The LGR5 DNA fragment was subsequently cloned into the XhoI and SmaI sites of the pCAG-AcGFP vector (Clontech, Mountain View, CA, USA) to generate the pCAG-AcGFP-LGR5 recombinant plasmid. A small interfering RNA expression vector that expresses the LGR5-specific short hairpin RNA (shRNA) was purchased from GenePharma Co., Ltd. (Shanghai, China). The LGR5 overexpression and shRNA vectors were transfected into SiHa and HeLa cells using the Lipofectamine 2000 reagent (Invitrogen) according to the manufacturer’s protocol. The transfected cells were treated with G418 (Calbiochem, La Jolla, CA, USA) for 3 weeks, and drug-resistant colonies were collected, expanded and identified.

### Flow cytometry analysis and FACS isolation of cells

The expression of LGR5 in cervical cancer cells and xenograft lines was measured using the Alexa Fluor 647 Rat anti-Human LGR5 (N-Terminal) antibody (562903, BD Biosciences, Franklin Lakes, NJ, USA) according to the manufacturer’s instructions. Flow cytometry was performed on a FACSCalibur or FACSAria Flow Cytometry System. The data were analyzed using the FlowJo software (Tree Star Inc., Ashland, OR, USA) or CellQuest program (BD Biosciences). Single cell suspensions were derived from xenograft tissue by mincing and digesting the tissue with 100 U/ml collagenase IV (GIBCO, Grand Island, NY, USA) in basal medium at 37 °C overnight.

### IHC

Formalin-fixed and paraffin-embedded tissue specimens were sliced into 4-mm sections that were then deparaffinized and hydrated. An endogenous antigen retrieval procedure was performed using citric acid buffer (10 mmol/l citrate buffer, pH 6.0). The slides were incubated with a rat monoclonal antibody raised against human LGR5 (1:50, Abnova, Taipei, Taiwan) overnight at 4 °C and then with secondary antibodies for 30 min at room temperature followed by diaminobenzidine development. All slides were examined under an Olympus-CX31 microscope (Olympus, Tokyo, Japan).

### *In vivo* tumor formation assays

The LGR5^+^ and LGR5^–^ cells were sorted, resuspended in 200 *μ*l of 1:1 PBS/Matrigel (BD Biosciences) and injected subcutaneously into the flanks of 6- to 8-week old female NOD/SCID mice (Charles River Laboratories, Wilmington, MA, USA); specifically, the left flank of the mouse received the LGR5^+^ cells, whereas the right flank received the LGR5^–^ cells. Engrafted mice were inspected twice per week by visual observation and palpation to determine the appearance of tumors. The tumor volume (*V*) was determined from the length (*a*) and the width (*b*) of the tumor, using the formula *V*=*ab*^2^/2. A portion of each tumor tissue was fixed in 10% formaldehyde and embedded in paraffin for IHC analysis. The frequency of tumorigenic cells (estimated with upper–lower limits) was calculated by FCS.

### Western blot

Cells were lysed in a lysis buffer (50 mM Tris-HCl, pH 7.4; 150 mM NaCl; 2 mM EDTA; 1% NP-40; and 0.1% sodium dodecyl sulfate) that contained a protease inhibitor cocktail (Complete Mini; Roche Diagnostics, Branchburg, NJ, USA). The membranes were incubated with antibodies against human LGR5 (1:200 dilution, Abnova, Taipei, Taiwan), ALDH1 (1:400 dilution, BD Biosciences, Franklin Lakes, NJ), BMI1 (1:500 dilutin, Millipore, Billerica, MA, USA), OCT4 (1:500 dilution, sc-9081, Santa Cruz, CA, USA), KLF4 (1:400 dilution, sc-20691, Santa Cruz, CA, USA), E-Cadherin (1:200 dilusion, sc-8426, Santa Cruz, Dallas, TX, USA), Vimentin (1:200 dilusion, sc-6260, Santa Cruz, Dallas, TX, USA), Snail (1:200 dilusion, sc-28199, Santa Cruz, Dallas, TX, USA) and β-actin (1:1000 dilution; Santa Cruz, CA, USA) at 4 °C overnight followed by incubation with horseradish peroxidase-conjugated secondary immunoglobulin G (IgG; Thermo Fisher Scientific, New York, NY, USA). The membranes were briefly incubated with an enhanced chemiluminescence reagent (Millipore) and then visualized on X-ray films.

### Tumorsphere culture

Cells were maintained in stem cell media consisting of DMEM/F12 basal media, N2 and B27 supplements (Invitrogen), 20 ng/ml human recombinant epidermal growth factor and 20 ng/ml basic fibroblastic growth factor (PeproTech Inc., Rocky Hill, NJ, USA). For the tumorsphere-formation assay, cells were plated at a density of 200 cells/well on 24-well ultra-low attachment plates or at a density of 1 cell/well on 96-well plates and maintained in stem cell medium. Tumorspheres that arose within 2 weeks were recorded. For serial tumorsphere-formation assays, the spheres were harvested, disaggregated with 0.25% trypsin/EDTA, filtered through a 40 *μ*m mesh and replated as described above. For each cell type, the experiments were performed in triplicate, and the spheres were counted by two individuals in a blinded fashion.

### Drug resistance and MTT assays

For the drug resistance assays, cells were plated in 96-well plates at a density of 10^4^ cells/well and allowed to recover overnight before initiating the drug treatments. The cells were exposed to various concentrations of cisplatin (0, 3, 6, 12, 24 or 48 *μ*g/ml) for 24 h, and the cell viability was measured. In other experiments, the cells were exposed to a constant concentration of cisplatin (3 *μ*g/ml) for 24, 48 or 72 h, and the cell viability was measured. Cell viability was assessed using the 3-(4,5-Dimethyl-1, 3-thiazol-2-yl)-2,5-diphenyl-2H-tetrazol-3-ium bromide (MTT; Sigma-Aldrich) assay. Following the manufacturer’s instructions, 20 *μ*l of MTT solution was added to 200 *μ*l of the culture media. The plates were then incubated for 4 h at 37 °C, and the optical density was measured at 490 nm.

### Soft agar cloning

Cells were counted, resuspended at 2 × 10^3^ cells/ml in medium (DMEM with 10% FBS and l-glutamine) containing 0.3% w/v agar (Bacto, Duckinson, Sparks, MD, USA) and overlaid onto a 30-mm dish containing a solidified bottom layer of 0.6% w/v agar in the same medium. After incubation for 10–15 days at 37 °C and 10% CO2, all dishes were stained by adding 1 ml/dish of 0.01% (w/v) crystal violet (Fronine, Taren Point, NSW, Australia), and the colonies were counted with a dissection microscope. The assays were performed in triplicate.

### Wound repair assays

Cells were plated in 24-well plates at 10^6^ cells/well in 1 ml of culture medium. Two days later, a wound was scratched in the adherent cell monolayers with an Eppendorf tip, and the medium was changed to DMEM supplemented with 1% FBS (Invitrogen). The wells were examined every two days, and photomicrographs were taken on a Nikon Eclipse Ti as described above. Wound width was measured on the photomicrographs, using the same area of the well for each measurement.

### Migration and invasion assays

Transwell chambers (Corning, Corning, NY, USA) equipped with 8-*μ*m-pore insets were used for the migration and invasion assays. For the migration assay, 4 × 10^4^ LGR5-overexpressing cells and control cells in serum-free medium were plated on uncoated insets and incubated for 12 h. Similarly, 8 × 10^4^ LGR5-knockdown cells and control cells in serum-free medium were plated on uncoated insets and incubated for 24 h. For the invasion assay, the insets were coated with 200 *μ*l of 1:3-diluted Matrigel (BD Biosciences), and 1 × 10^5^ cells were plated in the serum-free medium described above for an incubation period of 36 h. Similarly, 2 × 10^5^ LGR5-knockdown cells and control cells were plated in the serum-free medium described above for an incubation period of 48 h. Quantities of 600 ml of culture medium containing 20% FBS (Invitrogen) were added to the lower chamber. Non-invaded cells were removed, and the cells that were attached to the bottom of the membrane were fixed with 4% paraformaldehyde, stained with 5% crystal violet (Sigma-Aldrich) and counted at 200-fold magnification. These experiments were performed in triplicate.

### Statistical analysis

Statistical analyses were performed using the GraphPad Prism 5.01 software (La Jolla, CA, USA). In the comparisons of two groups, Student’s *t*-test was used to determine statistical significance. To examine differences among four groups, ANOVA was performed. Kaplan–Meier survival analysis was performed, and survival curve comparisons were performed using the log-rank (Mantel–Cox) test. *P*-values of ⩽0.05 were regarded as statistically significant.

### Ethics statement

This study has been conducted in accordance with the ethical standards of the Declaration of Helsinki and the national and international guidelines. It has been approved by the review board of the First Affiliated Hospital of Xi’an Jiaotong University.

## Publisher’s Note

Springer Nature remains neutral with regard to jurisdictional claims in published maps and institutional affiliations.

## Figures and Tables

**Table 1:** Tumorigenic capacity of LGR5^LGR5+^ and AcGFP^LGR5^^−^ cells in NOD/SCID mice from two cervical cancer cell lines

**Cell line**	**Subpopulation**	**Cell does**				**Tumor-initiating cell frequency (95% Interval)**	***P*-value**
		**10**^**4**^	**10**^**3**^	**10**^**2**^	**10**		
SiHa	SiHa-LGR5^LGR5+^	8/8	8/8	6/8	6/8	1:36 (1:79–1:16)	<0.001
	SiHa-AcGFP^LGR5−^	8/8	6/8	2/8	0/8	1:627 (1:1353–1:291)	
HeLa	HeLa-LGR5^LGR5+^	8/8	8/8	6/8	4/8	1:46 (1:100–1:22)	<0.001
	HeLa-AcGFP^LGR5−^	6/8	2/8	0/8	0/8	1:6,276 (1:13 541–1:2909)	
